# The Emerging Role of Hedgehog Signaling in Viral Infections

**DOI:** 10.3389/fmicb.2022.870316

**Published:** 2022-04-08

**Authors:** Yulin Zhou, Jinhua Huang, Boxin Jin, Su He, Yongfang Dang, Tiejun Zhao, Zhigang Jin

**Affiliations:** College of Chemistry and Life Sciences, Zhejiang Normal University, Jinhua, China

**Keywords:** hedgehog, GLI, virus, viral diseases, pathogenesis

## Abstract

The hedgehog (HH) signaling pathway is one of the key pathways that is indispensable for many developmental processes and postnatal tissue homeostasis. Dysregulated HH signaling could lead to developmental disorders and tumorigenesis in a variety of tissues *via* inherited or sporadic mutation, gene overexpression, and crosstalk with other signaling pathways. Recently, accumulating evidence has shown that HH signaling is targeted by viruses to facilitate viral transcription, immune evasion, and uncontrolled growth, leading to effective viral replication and pathogenesis. In this study, we will summarize recent advances in functional interaction between HH signaling and different types of viruses, particularly focusing on the pathological role of HH signaling in viral infections and related diseases.

## Hedgehog Signaling Pathway

### The Components of Hedgehog Signaling

Hedgehog (HH), originally named after the spiny appearance of the cuticle in the *Drosophila* mutant, was discovered as a segment polarity gene that governs segmental pattern along with other components of HH signaling (Nüsslein-Volhard and Wieschaus, 1980). Since then, numerous and rapid progress has been made in realizing the significance of HH signaling to a variety of processes during embryonic development, such as wing development in *Drosophila*, limb development, and neural patterning in vertebrates (Jiang and Hui, 2008; Briscoe and Thérond, 2013; Pak and Segal, 2016; Liu, 2019). The signaling mechanism is evolutionarily conserved ranging from *Drosophila* to human, although some divergences do exist between invertebrate and vertebrate, largely due to gene duplication-caused functional redundancy and extreme reliance on primary cilium as a signaling hub and suppressor of fused (SUFU) as a major repressor in vertebrate (Huangfu and Anderson, 2006; Varjosalo et al., 2006; Kuzhandaivel et al., 2014). The HH gene family encodes secreted proteins that undergo processing, release, spread, and reception, transducing signaling from HH-producing cells to HH-responding cells (Gallet, 2011). HH proteins are usually locally produced and form a concentration gradient that induces differential expression of HH target genes and directs tissue patterning. In mammals, three HH gene homologs have been identified, namely, sonic HH (SHH), Indian HH (IHH), and desert HH (DHH). SHH is broadly expressed and essential for the development of most regions in embryos, including limb and neural tube. IHH is more close to SHH and essential for the development of endochondral bone, whereas DHH regulates gonad development and myelination of peripheral nerves.

In vertebrates, the HH protein family triggers a signaling cascade that leads to alteration of the net balance between the activator form (GLI*^A^*) and the repressor form (GLI*^R^*) of the GLI family of zinc finger transcription factors, and ultimately regulates the specific expression of GLI*^A^*/GLI*^R^* target genes that defines cell identity (Jiang and Hui, 2008; Briscoe and Thérond, 2013; Pak and Segal, 2016; Liu, 2019). The twelve-span transmembrane protein, Patched 1 (PTCH1), is the major receptor of HH ligands that constitutively represses HH signaling. Smoothened (SMO) is a G protein-coupled receptor (GPCR)-like seven-span transmembrane co-receptor. However, SMO is a potent activator of the HH signaling pathway rather than the GPCR-related signaling pathway. The GLI family transcription factors, including GLI1, GLI2, and GLI3, serve as downstream effectors. GLI2 and GLI3 (GLI2/3) could either activate or suppress the transcription of HH target genes, depending on their GLI*^A^* or GLI*^R^* forms. In contrast, GLI1 serves as a transcriptional activator only and could be transcriptionally activated by HH signaling, forming a positive feedback to consolidate signaling strength and duration. SUFU is a major negative regulator of HH signaling in a vertebrate that functions between SMO and GLI proteins, mainly *via* sequestering GLI proteins in the cytoplasm and repressing transcriptional activity of GLI protein in the nucleus.

### Hedgehog Signal Transduction

**FIGURE 1:**
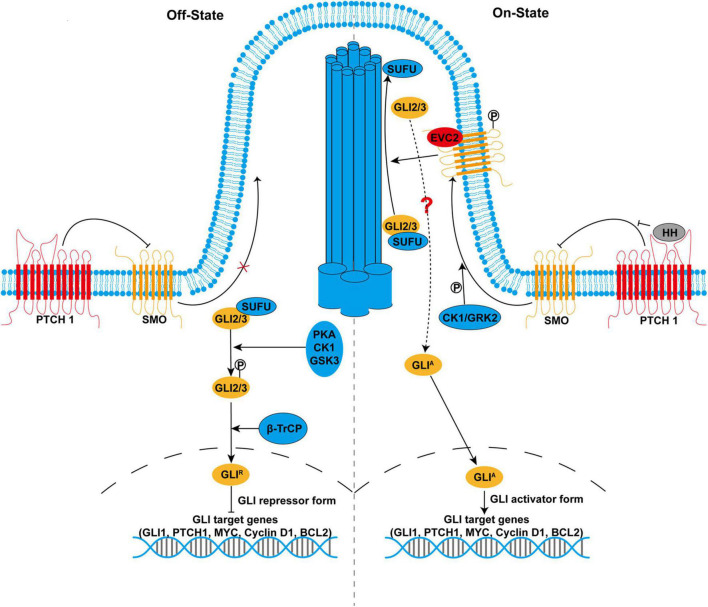
The hedgehog (HH) signaling pathway in mammals. In the absence of HH ligands **(left side)**, smoothened (SMO) is inhibited by primary cilium-localized Patched 1 (PTCH1). With the inactivated SMO, full-length GLI2 and GLI3 (GLI2/3) are retained in the cytoplasm by suppressor of fused (SUFU) and phosphorylated by protein kinase A (PKA), casein kinases 1 (CK1), and glycogen synthase kinase 3 (GSK3). Phosphorylated GLI2/3 are proteolytically processed by E3 ubiquitin ligase β-transducin repeat-containing protein, resulting in the production of the repressor form GLI*^R^*, which enters the nucleus to suppress the transcription of target genes. In the presence of HH ligands **(right side)**, binding of HH to PTCH1 derepresses SMO. SMO is phosphorylated by CK1 and GRK2 and translocates to the primary cilium. Activated SMO not only inhibits proteolytic processing of GLI2/3 but also promotes the recruitment of GLI2/3-SUFU complex to the tip of the primary cilium, leading to their dissociation and production of the activator form GLI*^A^*, a process that might involve Ellis-van Creveld/EVC2. GLI*^A^* enters the nucleus as transcription activators and promotes the transcription of target genes that are important for many cellular processes, including cell proliferation, differentiation, survival, and maintenance of stemness.

In the absence of HH ligands, PTCH1 is accumulated in the primary cilium where PTCH1 blocks SMO activity by preventing the translocation of SMO to primary cilium. In this condition, full-length GLI2/3 are retained in the cytoplasm by SUFU (Figure 1). Protein kinase A (PKA), casein kinases 1 (CK1), and glycogen synthase kinase 3 (GSK3) preferentially phosphorylate GLI2/3 when they exist as GLI2/3-SUFU complex. Hyperphosphorylated GLI2/3 are recognized by E3 ubiquitin ligase β-transducin repeat-containing protein (β-TrCP), resulting in proteolytic processing by the removal of C-terminal transactivation domain and conversion to the repressor form GLI*^R^*. Proteolytic processing of GLI2/3 is dependent on primary cilium as PKA is highly enriched in the base of primary cilium, which prime phosphorylation of GLI2/3 by GSK3 (Nozawa et al., 2013; Bangs and Anderson, 2017). Finally, GLI*^R^* enters the nucleus and suppresses the transcription of target genes. Alternatively, full-length GLI proteins could also be targeted for complete degradation *via* the ubiquitin-proteasome pathway, which accounts for the labile property of GLI proteins (Huntzicker et al., 2006; Wang et al., 2010; Liu, 2019).

In the presence of HH ligands, PTCH1 is internalized for degradation after binding to HH ligands, which derepresses SMO (Figure 1). SMO is phosphorylated by CK1 and G-protein-coupled receptor kinase 2 (GRK2) (Chen et al., 2011). Phosphorylated SMO translocates to primary cilium where SMO is activated. SMO is also activated by cholesterol modification in response to HH (Xiao et al., 2017). Active SMO inhibits PKA-mediated phosphorylation and proteolytic processing of GLI2/3. However, prevention of GLI*^R^* production alone is insufficient to fully activate HH signaling. Active SMO further promotes the recruitment of GLI2/3-SUFU complex to the tip of the primary cilium, where GLI2/3 are dissociated from SUFU and converted into the activator to form GLI*^A^ via* unknown modification. Although the precise signaling between SMO and GLI2/3 remains largely unclear, studies have shown that phosphorylated SMO forms a complex with two ciliary proteins, Ellis-van Creveld (EVC)/EVC2 upon HH stimulation. EVC/EVC2 activates HH signaling downstream of SMO by facilitating cilia localization of GLI2/3-SUFU complex (Dorn et al., 2012; Yang et al., 2012). Therefore, SMO simultaneously relieves the repression of GLI protein by PKA and SUFU, leading to a switch from GLI*^R^* to GLI*^A^*. Finally, GLI*^A^* enters the nucleus as transcription activators to promote the transcription of target genes, including *GLI1*, *PTCH1*, *MYC*, *Cyclin D1*, and *BCL2*. Meanwhile, GLI*^A^* becomes very labile due to Speckle-type POZ protein (SPOP)-mediated degradation, which might be responsible for the termination of HH signaling (Wang et al., 2010; Shi et al., 2014).

A list of novel components of HH signaling that function in a general or context-dependent manner is continuously growing, such as G protein-coupled receptor 161 (GPR161), inositol polyphosphate 5-phosphatase E (INPP5E), and karyopherin β2 (KAPβ2), SLIT and NTRK-like protein-5 (SLITRK5) (Mukhopadhyay et al., 2013; Garcia-Gonzalo et al., 2015; Han et al., 2017; Sun et al., 2021). We have also found that Daz interacting protein 1 (Dzip1) and RUN and SH3 domain-containing 2 (Rusc2) are involved in the regulation of HH signaling and are required for proper eye development in *Xenopus* (Jin et al., 2011, 2016; Schwend et al., 2013). In addition to the canonical HH signaling, non-canonical HH signaling that GLI proteins are cross-activated by other signaling pathways independent of HH ligands or SMO may also occur, especially in pathological conditions (Pietrobono et al., 2019; Sigafoos et al., 2021).

## The Role of Hedgehog Signaling

### The Physiological Role of Hedgehog Signaling

Owing to the diverse sets of target genes, HH signaling is involved in the control of many cellular responses, including cell proliferation, differentiation, and survival (Jiang and Hui, 2008; Briscoe and Thérond, 2013; Pak and Segal, 2016; Liu, 2019). For example, a concentration gradient of HH in the developing neural tube regulates neural patterning through transcriptional activation of *NK6 homeobox 1* (*NKX6.1*), *oligodendrocyte transcription factor 2* (*OLIG2*), and *NKX2.2*. HH signaling is required for the maintenance of stem or progenitor cells in embryonic and adult tissues *via* targeting cell cycle genes *MYC* and *Cyclin D1* and stemness-associated gene *NANOG* and *SOX2* (Ahn and Joyner, 2005; Po et al., 2010; Briscoe and Thérond, 2013; Iriana et al., 2021). HH signaling promotes cell survival *via* targeting the anti-apoptotic gene *BCL2* (Bigelow et al., 2004). In addition, HH signaling maintains the integrity and function of the blood-brain barrier (BBB) *via* its effector *Netrin-1* (Alvarez et al., 2011; Podjaski et al., 2015). As HH signaling is substantially involved in embryonic development and adult tissue homeostasis, it must be under tight control. Not surprisingly, aberrant HH signaling caused by genetic or somatic mutations and abnormal expression of HH-related proteins is closely associated with a lot of human diseases, such as developmental disorders and cancer.

### Dysregulated Hedgehog Signaling in Developmental Disorders

Dysregulated HH signaling is responsible for many types of developmental disorders. Holoprosencephaly, a common birth defect characterized by incomplete division of the forebrain into two hemispheres, is usually caused by reduced HH signaling due to deletion or mutation in *SHH* and downstream genes (Nieuwenhuis and Hui, 2005; Loo et al., 2021). Compromised HH signaling impairs the eye separation process and induces cyclopia as seen in severe cases of holoprosencephaly, whereas increased HH signaling reduces the eye size (Lee et al., 2014; Jin et al., 2016). Consistently with its important role in limb development, GLI3 is a causative gene mutated in Greig cephalopolysyndactyly syndrome that is characterized by limb defects. Mutations in ciliary genes that affect the proper structure and function of primary or motile cilium could elicit a group of genetic disorders known as ciliopathies, such as Bardet-Biedl syndrome, Joubert syndrome, and Meckel-Gruber syndrome (Andreu-Cervera et al., 2021; Loo et al., 2021). Since primary cilium is a necessary signaling hub to integrate HH signaling cascade, disruption of HH signaling also underlies the pathogenesis of ciliopathies.

### Dysregulated Hedgehog Signaling in Cancer

The relationship between HH signaling and cancer was initially discovered in nevoid basal cell carcinoma syndrome (NBCCS, also known as Gorlin syndrome), a rare developmental disorder that is prone to develop into BCC and medulloblastoma (Hahn et al., 1996). The most common causes of NBCCS are loss-of-function mutations in two repressors of HH signaling, *PTCH1*, and *SUFU*, leading to constitutive activation of HH signaling (Onodera et al., 2020). Elevated HH signaling is a hallmark of many types of human cancer, including BCC and medulloblastoma (Pak and Segal, 2016; Wu et al., 2017; Sigafoos et al., 2021). Loss-of-function mutations in PTCH, SUFU, and gain-of-function mutations in SMO are usually found in the patients of BCC and medulloblastomas. These mutations aberrantly activate HH signaling bypassing HH ligands and the tight regulation of signaling cascade.

In contrast, ligand-dependent activation of HH signaling usually occurs in other types of cancer that rarely contain oncogenic mutations, such as prostate cancer, gastric cancer, and gliomas. In these conditions, elevated HH signaling supports the proliferation and survival of cancer cells in an autocrine way where cancer cells act as both HH-producing and HH-responding cells. Alternatively, a pathway is activated in a paracrine way where HH ligands secreted by cancer-surrounding stromal cells stimulate HH signaling in cancer cells, or HH ligands secreted by cancer cells stimulate HH signaling in stromal cells which in turn release cancer-supporting signals (Pak and Segal, 2016; Wu et al., 2017; Sigafoos et al., 2021). HH signaling also drives cancer initiation and progression by the maintenance of cancer stem cells in an undifferentiated and proliferative state (Cochrane et al., 2015).

## Hedgehog Signaling and Immunomodulation

Consistent with the important role of HH signaling in development and homeostasis, selective populations of immature thymocytes and peripheral mature T cells are responsive to HH ligands, and activation of HH signaling is required for fetal and adult T-cell development and peripheral T-cell activation (Crompton et al., 2007; de la Roche et al., 2013). In contrast, HH signaling promotes central nervous system (CNS) immune quiescence by counterbalancing inflammatory events (Alvarez et al., 2011). Thus, HH signaling positively or negatively influences immune response in a context-dependent manner. In recent years, numerous studies have emerged that HH signaling is involved in the evasion of anticancer and antiviral immune response in cancer cells and virus-infected cells, respectively (Hanna and Shevde, 2016; Iriana et al., 2021). It appears that most pathological conditions preferentially switch the outcome of HH signaling toward the suppression of host immunity, especially in cancer and virus-related diseases.

Using T-cell-specific GLI2 transgenic mice, Furmanski et al. (2015) demonstrated that transcription activated by GLI2*^A^* in T cells attenuates T-cell signaling and T-cell activation induced by T-cell receptor (TCR) stimulation. GLI2*^A^* impairs the activation of pro-inflammatory AP-1 and NF-κB signaling. In contrast, GLI2*^R^*-mediated transcription repression increases NF-κB activity following TCR stimulation. Thus, HH signaling might skew pro-inflammatory immune responses during inflammation and tissue repair. Indeed, activation of HH signaling increases CD4^+^ regulatory T-cells (Treg) populations and their immunosuppressive function by activating transforming growth factor-β (TGF-β) production, thereby preventing skin inflammation in the mouse model of atopic dermatitis (Papaioannou et al., 2019). Studies in SMO conditional knockout mice have shown that HH signaling deficiency in CD4^+^ T cells exacerbates brain-brainstem-cerebellum neuroinflammation (Benallegue et al., 2021). Mechanistically, HH signaling antagonizes CD4^+^ T-cells-driven neuroinflammation by limiting their production of inflammatory cytokines at the transcriptome level. Notably, intestinal epithelium-derived IHH signals to the stromal cells and maintains immune tolerance of the intestine through suppression of stromal C-X-C motif chemokine ligand 12 (CXCL12), whereas loss of IHH induces a rapid immune response characterized by upregulation of stromal CXCL12 and filtration of immune cells (Westendorp et al., 2018). In the context of the tumor microenvironment, tumor-derived SHH executes immunosuppressive and tumor-supporting functions by promoting Krüppel-like factor 4 (KLF4)-mediated polarization of M2 tumor-associated macrophages (TAMs) and upregulation of PD-L1 expression in TAMs (Petty et al., 2019, 2021). In BCC and medulloblastoma, GLI1 and GLI2 (GLI1/2) directly activate the transcription of suppressor of cytokine signaling 1 (SOCS1) and reduce signal transducer and activator of transcription 1 (STAT1) phosphorylation, imposing a negative effect on Interferon (IFN)-γ/STAT1 signaling and anticancer immunity (Laner-Plamberger et al., 2013). Collectively, HH signaling is deployed by a subset of immune cells and cancer cells to suppress host immunity by expanding Treg population, interfering with immune signaling, and production of cytokines or chemokines.

**FIGURE 2:**
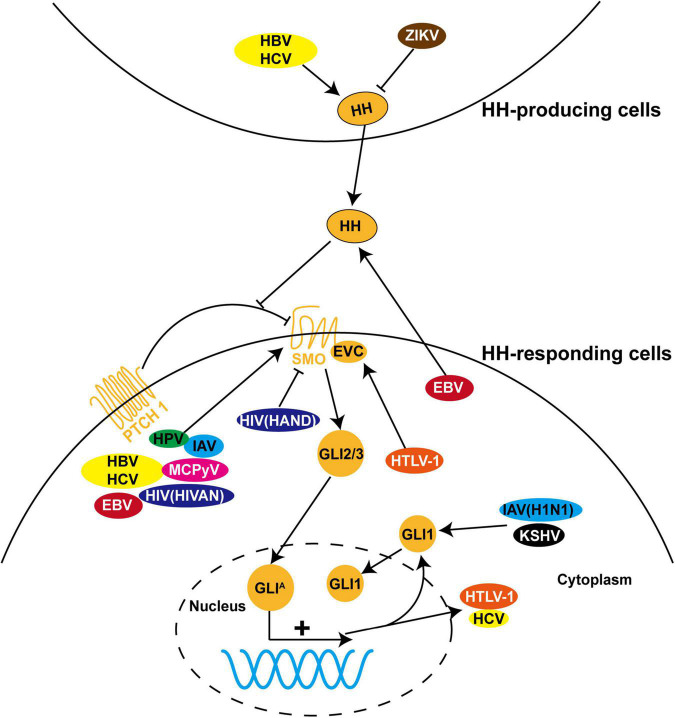
The role of HH signaling in viral infections. HH pathway is dysregulated during infection with a variety of viruses, including hepatitis B virus (HBV), hepatitis C virus (HCV), Epstein-Barr virus (EBV), human papillomavirus (HPV), human immunodeficiency virus (HIV), human T-cell leukemia virus type I (HTLV-1), influenza A virus (IAV), Merkel cell polyomavirus (MCPyV), Zika virus (ZIKV), and Kaposi’s sarcoma-associated herpesvirus (KSHV). Most of these viruses induce pathological processes by regulating the expression, protein stability, and subcellular localization of GLI proteins and other components of HH signaling in HH-responding cells. In contrast, dysregulation of HH signaling is triggered in HH-producing cells by ZIKV and in both cells by HBV and HCV. In addition, HTLV-1 and HCV employ HH signaling to facilitate viral transcription and replication.

As HH signaling is substantially involved in cell proliferation, survival, and immunomodulation, it becomes a preferred target for a variety of viruses to evade antiviral immunity and support viral life cycles (Figure 2). In most virus-related cancers, HH signaling is abnormally activated by viral proteins to drive the tumor initiation process and metastatic cascade (Hanna and Shevde, 2016; Iriana et al., 2021; Trivedi et al., 2021). Inspired by the elegant review of Smelkinson that sheds light on HH signaling as a pathogenic target (Smelkinson, 2017), in the following sections, we will overview the recent progress on the functional interaction between HH signaling and viruses, in the order of virus types.

## Hedgehog Signaling in Viral Infections

### Hepatitis Viruses

Hepatocellular carcinoma (HCC) is one of the leading causes of cancer deaths. About 80% of HCC incidence is associated with chronic infection of hepatitis B virus (HBV) and hepatitis C virus (HCV) (Arzumanyan et al., 2013). HH signaling is maintained at low activity in normal mature hepatocytes but re-activated in the liver of patients with HCC, as illustrated by elevated levels of SHH, SMO, GLI2, and HH target genes in HCC tissues compared to the adjacent normal tissues (Huang et al., 2006; Sicklick et al., 2006; Crompton et al., 2007; Pereira Tde et al., 2010). Chronic infection of HBV or HCV increases the hepatic production of SHH and IHH. Accordingly, the populations of HH-responding cells, including liver myofibroblasts and activated endothelial cells, are expanded, which promotes liver fibrosis and HCC (Pereira Tde et al., 2010). In addition, the expression of SMO directly correlates with HCC tumor size (Sicklick et al., 2006). Virus-induced activation of HH signaling contributes to multiple aspects during the pathogenesis of HCC, including maintenance of cancer stem cells, initiation of epithelial-mesenchymal transition (EMT), metastasis, and drug resistance (Dimri and Satyanarayana, 2020; Zhou et al., 2020; Ding et al., 2021).

As a major cause of HCC, HBV is a small, double-stranded DNA virus but similar to retroviruses in the viral life cycle and genome organization. Integration of viral DNA into the host genome is an important step during the pathogenesis of HBV-induced HCC. HBV integrations are more abundant in HCC tissues than in adjacent normal tissues and HBV integration-targeted genes are significantly enriched in the HH signaling pathway (Yang et al., 2018). However, the effect of HBV integrations on HH signaling and its pathological significance remain to be determined. HBV-encoded X protein (HBx) is a central pathogenic factor in HCC, which plays an indispensable role in signal transduction, cell cycle progress, protein degradation, apoptosis, and genetic stability (Arzumanyan et al., 2013). Further investigation revealed that HBx accounts for HBV-induced activation of HH signaling. HBx promotes protein stability and nuclear translocation of GLI1/2 by direct protein-protein interaction, concomitant with increased transcriptional activity of GLI1/2 (Kim et al., 2011). HBx expression causes upregulation of HH activity in the liver of patients with HCC infected with HBV and develops HCC phenotypes in HBx transgenic mice (Arzumanyan et al., 2012). Moreover, inhibition of HH signaling by treatment with SMO inhibitor GDC-0449 attenuates cell growth and migration in HCC cell lines and delays tumor development in HBx transgenic mice and xenograft-bearing nude mice. In contrast, GDC-0449 merely affects tumor growth in the nude mice bearing HBx negative HCC cells. Taken together, these findings demonstrate that HBV-mediated pro-tumor function is dependent on HBx-induced activation of HH signaling.

HCV is another leading cause of liver fibrosis and HCC. Unlike HBV, HCV is a positive sense, single-stranded RNA virus. Infection of hepatocytes with HCV significantly increases the expression of SHH mRNA (Pereira Tde et al., 2010). HCV-permissive Huh7.5 HCC cells exhibit enhanced HH activity and mesenchymal identity as compared to parental Huh7 cells that are less permissive for HCV replication (Choi et al., 2011). Interestingly, HCV replication is facilitated in Huh7 cells treated with SHH ligand and SMO agonist SAG, whereas HCV replication is compromised in Huh7.5 cells treated with SMO inhibitor cyclopamine and GDC-0449. In addition, sera of HCV-infected patients increase GLI2 expression and its nuclear accumulation, leading to pro-fibrotic effects, which are antagonized by GLI 1/2 inhibitor GANT 61 (Granato et al., 2016). Collectively, these studies have not only revealed the pathological significance of HH signaling in HCV-induced liver fibrosis and HCC, but also introduced HH pathway inhibitors as a potential therapeutic strategy. However, further investigation is required to address which and how viral proteins target HH signaling for activation during HCV pathogenesis.

Hepatitis D virus (HDV) is a small RNA virus that requires HBV for infection and replication (Farci et al., 2021). Compared to HBV and HCV, the pathogenic process of HDV in HCC is largely unknown. A recent study of transcriptomic profiling identified enrichment of upregulated transcripts of HH signaling pathway in HDV-associated HCC, indicating that activation of HH signaling might be a common mechanism that HBV, HCV, and HDV have evolved to drive HCC progression (Diaz et al., 2018; Farci et al., 2021).

### Epstein-Barr Virus

Epstein-Barr virus (EBV) is a member of the herpesvirus family that contributes to about 1.5% of all cases of human cancer, including Hodgkin’s lymphoma, nasopharyngeal carcinoma (NPC), and gastric cancer (Farrell, 2019). Dysregulation of the HH signaling pathway plays an important role in the development of EBV-related NPC. A study of microarray profiling found that the expression levels of SHH ligand and HH target genes are upregulated in EBV-infected NPC cell lines and tissues (Port et al., 2013). EBV activates HH signaling in epithelial cells through autocrine induction of SHH ligand, leading to increased expression of stemness-associated genes. This process is recapitulated by overexpression of EBV latent membrane protein 1 (LMP1) and LMP2A but blocked by treatment of GLI1/2 inhibitor GANT 58 or GANT 61, indicating that EBV drives NPC progression through LMP1- and LMP2A-mediated activation of HH signaling. Consistent with dysregulation of HH signaling in EBV infection, HH signaling was identified in the genomic mutation landscape of EBV-positive NPC (Tu et al., 2018). Computational analysis predicted 175 functional interactions between EBV proteins and the known components of HH signaling (Mei and Zhang, 2016).

Abnormal HH signaling is also associated with EBV-related gastric cancer. Downregulation of the human leukocyte antigen (HLA) is one of the common strategies deployed by cancer cells and viruses to evade host immune response. EBV-encoded LMP2A protein downregulates the expression of HLA Class I in gastric cancer cells (Deb Pal and Banerjee, 2015). Mechanistically, LMP2A activates HH signaling in EBV-infected gastric cancer cells, whereas blocking HH signaling by knockdown of GLI1 reverses LMP2A-mediated downregulation of HLA. Thus, EBV suppresses HLA expression to evade host immunity *via* LMP2A-mediated activation of HH signaling. Liang et al. (2014) found that the *IHH* gene is transcriptionally downregulated by EBV-associated hypermethylation in gastric cancer cells infected with EBV. Downregulation of IHH increases cell growth and colony formation ability, suggesting a tumor-suppressive potential of IHH in EBV-related gastric cancer (Liang et al., 2014). These studies imply that HH signaling participates in the occurrence and development of EBV-induced diseases.

### Human Papillomavirus

Human papillomavirus (HPV) is a group of related double-stranded DNA viruses known as the primary cause of cervical cancer. HPV 16 and HPV 18 are responsible for at least 70% of cases of cervical cancer, largely dependent on two oncogenes E6 and E7 (Pal and Kundu, 2019). An early link between HH signaling and HPV was discovered in studies, showing that components or target genes of HH signaling are elevated in E7 transgenic mice and patients with cervical cancer (Chaudary et al., 2012; Ibarra Sierra et al., 2012). In agreement with this, inhibition of HH signaling by cyclopamine or GANT 61 reduces the proliferation and survival of cervical cancer cells (Samarzija and Beard, 2012).

A recent study found that GLI1 and E6 stimulate their transcriptional expression reciprocally, resulting in a high level of GLI1 and E6 in cervical cancer stem cells (Vishnoi et al., 2016). The cooperation between GLI1 and E6 maintains the population and stemness of cervical cancer stem cells. Accordingly, simultaneous inhibition of SMO and E6 results in an addictive anticancer effect. Thus, the crosstalk between GLI1 and E6 might be a strategy utilized by HPV to select a subset of cervical cells for immortalization. The crosstalk between HH signaling and E6 was further confirmed in the mouse model of cervical cancer (Rojo-León et al., 2019). E6/E7 transgenic mice exhibit increased HH signaling activity, which is potentiated in the cervix by treatment of estradiol. E6/E7 oncogenes might cooperate with estradiol to promote uncontrolled growth of the cervix by synergistic activation of HH signaling. Due to the toxicity of SMO inhibitor cyclopamine, a low dose of cyclopamine is used to treat E6/E7 transgenic mice but does not cause an obvious effect on the cervix. Another SMO inhibitor itraconazole reduces growth at an early stage of cervical carcinogenesis but fails to decrease GLI1 activity. Nevertheless, activation of non-canonical HH signaling or canonical HH signaling but downstream of SMO (e.g., loss-of-function mutation of SUFU and gain-of-function mutation of GLI1) might occur during the multistep process of cervical carcinogenesis. If this is the case, GLI1 inhibitor rather than SMO inhibitor would be more suitable for the development of cervical cancer therapy. In conclusion, activation of HH signaling underlies the pathogenesis of HPV-induced cervical cancer. However, the application of HH signaling inhibitor as a candidate therapy for cervical cancer remains to be further explored.

### Human Immunodeficiency Virus

Human immunodeficiency virus (HIV) infection is linked to the suppression of protective immune responses and causes acquired immune deficiency syndrome (AIDS). An early study has shown that the expression of immunosuppressive cytokine TGF-β1 is elevated after HIV-1 infection and leads to the development of induced Treg (Furler and Uittenbogaart, 2012). As a main downstream transcription factor of the HH signaling pathway, GLI2 protein enhances the expression of TGF-β1 in naïve CD4^+^ T cells through activating the human TGF-β1 promoter activity. The HIV-1 encoded viral protein Tat has been implicated as the inducer of TGF-β1 in both *in vitro* and *in vivo*. The identified interaction between Tat and GLI2 raises the possibility that they might synergistically activate TGF-β1 transcription after HIV infection and contribute to immune suppression.

The HIV-associated neurocognitive disorder (HAND) is characterized by the recruitment of infected leukocytes into CNS *via* damaged BBB. As mentioned earlier, HH signaling is essential for the maintenance of BBB integrity (Alvarez et al., 2011). Impaired HH signaling in the CNS correlates with HIV-induced loss of BBB function and neurological injury, whereas re-activation of HH signaling by SAG reduces the viral load in the CNS and rescues BBB integrity and neuroprotection in HIV-infected humanized mice (Singh et al., 2016, 2017). Bohannon et al. (2019) confirmed the disruption of BBB in infected patients with HIV and simian immunodeficiency virus (SIV)-infected rhesus macaques. However, they found the persistent presence of SHH at the BBB. Netrin-1, an important downstream effector of HH signaling, is produced by brain pericytes to fortify BBB (Podjaski et al., 2015). Despite the strong presence of SHH, Netrin-1 and its cellular source pericytes are absent in encephalitic lesions. Therefore, these studies suggest that impaired HH signaling in pericytes might occur downstream of SHH ligand, which contributes to HIV-induced BBB breakdown and neuropathogenesis in HAND.

The HIV-induced EMT of podocytes is an important mechanism of HIV-associated nephropathy (HIVAN). HH signaling is activated in human podocytes infected with HIV and the mouse model of HIVAN (Lan et al., 2017). Treatment with recombinant SHH or overexpression of GLI1 significantly enhances EMT, while blocking HH signaling by GLI1/2 inhibitor GANT 58 attenuates HIV-induced EMT in podocytes, indicating that HIV-induced EMT and kidney injury are dependent on activation of HH signaling. Taken together, these studies reveal that HH signaling plays a key role in the development of HIV-related diseases.

### Human T-Cell Leukemia Virus Type 1

Adult T-cell leukemia (ATL) is a peripheral T-lymphocyte malignancy caused by infection of the retrovirus human T-cell leukemia virus type I (HTLV-1). HTLV-1 bZIP factor (HBZ) and Tax play a critical role in leukemogenesis of ATL, and double transgenic mice of HBZ and Tax in CD4^+^ T cells develop T-cell lymphoma (Zhao et al., 2014; Nosaka and Matsuoka, 2021). HTLV-1-encoded Tax is a potent transcription activator of viral genes dependent on its interaction with a series of host proteins, such as GLI2 (Tanimura et al., 1998; Dan et al., 1999). GLI2 and cyclic AMP response element-binding protein (CREB) form a complex with Tax in the long terminal repeats of HTLV-1 and facilitate Tax-mediated activation of viral transcription.

Tax extensively crosstalks with cellular signaling pathways to subvert cellular functions and suppresses host defense. EVC1 and EVC2, positive modulators of HH signaling that act downstream of SMO, are found to be aberrantly overexpressed in ATL and HTLV-1-infected cells (Takahashi et al., 2014). The upregulation of EVC1 and EVC2 is mediated by Tax *via* epigenetic modification at the EVC loci. Knockdown of EVC1, EVC2, GLI1 or GLI2, or treatment with GLI1/2 inhibitor GANT 61 induces apoptosis of ATL cells, indicating that EVC1/2-mediated activation of HH signaling supports the survival of ATL cells. Thus, HH signaling plays a vital role in ATL pathogenesis and represents a potential therapeutic target of ATL.

### Influenza A Viruses

Influenza A viruses (IAVs) are the causative pathogens of seasonal influenza epidemics that affect the upper and lower respiratory tract and represent a wide spectrum of subtypes based on surface proteins hemagglutinin (H) and neuraminidase (N). IAV-encoded non-structural protein NS1 is highly expressed in infected cells. NS1 activates HH signaling *via* direct interaction with transcription factor Ci in *Drosophila* (Smelkinson et al., 2017). The A122 residue of NS1 is critical for establishing interaction and regulation of Ci. Expression of NS1 also exerts an HH-modulating activity in human lung epithelial cells and infected mouse lungs. Infection of mice with IAV strongly induces the expression of HH target genes PTCH1 and BMP2 as well as cytokines CXCL10 and IL6 in an autocrine fashion. Surprisingly, IAV with NS1 A122V mutation induces the higher expression of PTCH1 and IL6 and potentiates the lethality in mice, probably due to cytokine storm elicited by other viral factors in the context of virus-infected mice. Thus, NS1 displays a conserved role cross-species in targeting HH signaling for modulation but a divergent output of A122V mutation that might alter influenza virulence.

Influenza is characterized by the destruction of alveolar epithelial and endothelial, which involves IAV-induced activation of non-canonical HH signaling followed by disruption of the epithelial junctions (Ruan et al., 2020). GLI1 is cross-activated by MAPK and PI3K pathways in human lung cells and mice infected with the H1N1 strain. Increased expression of GLI1 induces the expression of SNAIL and SLUG, which downregulate junction proteins Occludin and ZO-1 and increase paracellular permeability of alveolar barrier. GLI1/2 inhibitor GANT 61 specifically suppresses the expression of SNAIL and SLUG in IAV-infected cells but not in uninfected cells. Consequently, the expression of junction proteins and integrity of the alveolar barrier are restored. Collectively, non-canonical activation of GLI1 plays a critical role in disruption of the alveolar barrier during the pathogenesis of IAV.

### Merkel Cell Polyomavirus

Merkel cell carcinoma (MCC) is aggressive neuroendocrine skin cancer and its primary cause is Merkel cell polyomavirus (MCPyV). It has been reported that the expression of HH target genes GLI1 and PTCH1 is increased in MCC (Brunner et al., 2010; Gambichler et al., 2021). SHH level is significantly elevated in MCPyV-positive MCC than in MCPyV-negative MCC, indicating that MCPyV activates HH signaling in MCC (Kuromi et al., 2017). MCPyV-encoded small T and large T antigens are recognized as the main driver for the carcinogenesis of MCC. While expression of T antigens or GLI1 alone only induces differentiation of human keratinocytes to the progenitors of Merkel cells, a combination of T antigens and GLI1 gives rise to a mature phenotype of Merkel cells (Kervarrec et al., 2020). As the executor of HH signaling, GLI1 initiates Merkel cell differentiation by induction of SOX2. This study suggests that activation of HH signaling contributes to the initial origin establishment of MCC cells but might be lost during the following tumor development.

### Zika Virus

Infection of Zika virus (ZIKV) increases the incidence of microencephaly in fetuses and Guillain-Barre syndrome in adults. The close association between HH signaling with neurodevelopmental disorders raises the possibility that HH signaling might be implicated in ZIKV-induced microencephaly. Using the developing chicken brains as an animal model, Thawani et al. (2018) found that signaling centers are more sensitive to ZIKV infection. ZIKV strongly infects the midbrain floor plate and causes increased apoptosis and decreased proliferation of SHH-producing cells. Insufficient production of SHH impairs the expression of HH target genes in the adjacent ventral midbrain and consequent neural patterning. Thus, ZIKV-induced deficiency of SHH disrupts midbrain development in a paracrine way, resulting in reduced size of the midbrain.

### Kaposi’s Sarcoma-Associated Herpesvirus

Kaposi’s sarcoma-associated herpesvirus (KSHV), also known as human herpesvirus 8 (HHV8), is a double-stranded DNA virus linked to Kaposi’s sarcoma (KS) and primary effusion lymphoma (PEL). Recently, Asha et al. (2020) discovered a significant increase of GLI1 expression in KS skin tissues and PEL cells infected with KSHV, which might be triggered in an HH ligands independent, but AMPK-dependent, manner. GLI1/2 inhibitor GANT 61 regresses the KS tumor formation. Although the precise mechanism involving the viral role in HH signaling activation remains to be investigated, this study uncovered the importance of non-canonical HH signaling to KSHV pathogenesis and its potential as a therapeutic target.

## Therapeutic Implication

**FIGURE 3:**
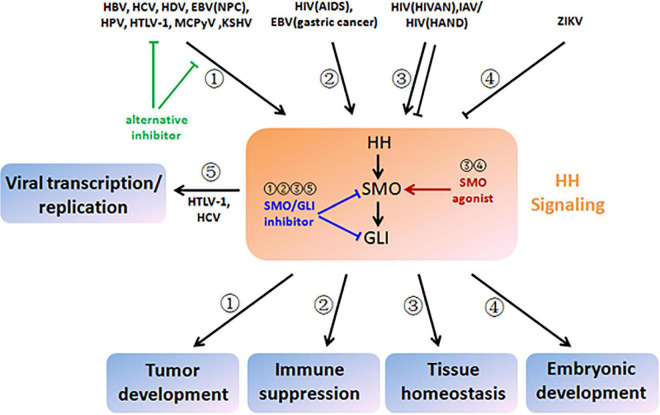
Therapeutic strategies based on viral modulation and pathological outcome of HH signaling. HBV, HCV, HDV, EBV (nasopharyngeal carcinoma), HPV, HTLV-1, MCPyV, and KSHV aberrantly activate HH signaling and drive multistep tumor development (pathway ➀). HIV (acquired immune deficiency syndrome) and EBV (gastric cancer) also activate HH signaling, which contributes to immune suppression (pathway ➁). HIV (HIV-associated nephropathy), IAV, and HIV (HIV-associated neurocognitive disorder) disturb tissue homeostasis by induction of either increased or decreased HH signaling (pathway ➂). ZIKA infection results in disruption of embryonic development by inactivation of HH signaling (pathway ➃). HTLV-1 and HCV hijack HH signaling and facilitate viral transcription and replication (pathway ➄). To reverse viral modulation and restore to the normal activity of HH signaling, SMO/GLI inhibitors could be applied to the scenarios of ➀, ➁, ➂, and ➄ (blunt arrows in blue), while SMO agonists could be applied to the scenarios of ➂ and ➂ (sharp arrows in red). Alternatively, chemical inhibitors could also target viral proteins that crosstalk with HH signaling or their interactions for blockage (blunt arrows in green).

Mechanistic insight into HH signaling and the discovery of its chemical modulators are mutually beneficial and co-developing. Since cyclopamine from the plant *Veratrum californicum* was identified as the teratogen responsible for cyclops lambs in 1968 and as the first HH signaling inhibitor in 1998, searching chemical modulators of HH signaling for the therapeutic purpose have attracted numerous research attentions (Lee et al., 2014). These efforts lead to the discovery of current food and drug administration (FDA)-approved SMO inhibitors GDC-0449 (Vismodegib), LDE225 (Sonidegib), and GLI inhibitor arsenic trioxide for cancer treatment, and even more are under investigation (Wu et al., 2017). As HH signaling is also dysregulated during infections of a wide range of viruses (Figure 2), a new and promising application of these chemical modulators is to restore dysregulated HH signaling in virus-related diseases. However, caution should be exercised especially in those non-life-threatening diseases due to possible disruption of tissue homeostasis. Importantly, appropriate chemical modulators, including SMO inhibitor, GLI1/2 inhibitor, and SMO agonist, should be carefully selected according to the diversity and complexity of viral modulations and pathological outcomes of HH signaling (Table 1 and Figure 3, blue and red arrows). An alternative strategy that could be expected is to target viral proteins responsible for modulation of HH signaling or their interactions for blockage (Figure 3, green arrows).

**TABLE 1:** The pathological role of hedgehog signaling in viral infections and the potential therapeutic strategies.

Viral modulation of HH signaling	Virus	Disease	Pathological outcome	Therapeutic strategy	References
Signaling activation (canonical)	HBV	Viral hepatitis, liver fibrosis, cirrhosis and HCC	Viral replication, tumor development	SMO inhibitor or GLI inhibitor	Pereira Tde et al., 2010; Arzumanyan et al., 2012
	HCV				Choi et al., 2011; Granato et al., 2016
	HDV				Diaz et al., 2018
	EBV	NPC	Tumor development		Port et al., 2013
		Gastric cancer	Immune suppression		Deb Pal and Banerjee, 2015
	HPV	Cervical cancer	Tumor development		Vishnoi et al., 2016; Rojo-León et al., 2019
	HIV	AIDS	Immune suppression		Furler and Uittenbogaart, 2012
		HIVAN	EMT induction		Lan et al., 2017
	IAV	Influenza	Cytokine production		Smelkinson et al., 2017
	MCPyV	MCC	Induction of MCC cell origin		Kervarrec et al., 2020
Signaling activation (non-canonical or downstream of SMO)	HTLV-1	ATL	Viral transcription, cancer cell survival	GLI inhibitor	Tanimura et al., 1998; Takahashi et al., 2014
	IAV(H1N1)	Influenza	Destruction of alveolar barrier		Ruan et al., 2020
	KSHV	KS, PEL	Tumor development		Asha et al., 2020
Signaling reduction	HIV	HAND	BBB breakdown and neuropathogenesis	SMO agonist	Singh et al., 2016; Bohannon et al., 2019
	ZIKV	Microencephaly	Disruption of midbrain development		Thawani et al., 2018

## Conclusion and Perspective

The HH signaling regulates numerous biological processes during embryonic development and postnatal tissue homeostasis. Dysregulation of HH signaling is the important driving force during the initiation and progression of a variety of diseases. In general, HH signaling is aberrantly activated in cancer while reduced in developmental disorders. Recently, accumulating studies have extended the pathological role of HH signaling to viral infections and their related diseases. Similarly, most viral infections that lead to cancer usually activate cellular HH signaling. Viral infections that lead to developmental disorders tend to decrease HH signaling activity, as exampled by ZIKV-induced microencephaly (Thawani et al., 2018). During viral-induced disruption of tissue homeostasis, both positive (IAV-induced disruption of the alveolar barrier) and negative (HIV-induced HAND) modulations of HH signaling are observed (Singh et al., 2016; Ruan et al., 2020). Notably, as a viral niche in host tissues might be different or keep changing after the initial infection, viral modulation of HH signaling and its underlying mechanism might be altered concomitantly. This is the case for HIV which activates HH signaling in the kidney to enhance EMT while inactivating HH signaling in the brain to impair BBB (Singh et al., 2016; Lan et al., 2017). This could even occur in the same viral disease but at different stages, which is implicated by MCPyV-related MCC (Kervarrec et al., 2020). Collectively, these recent advances have not only unveiled the emerging role of HH signaling in viral infections but also evoked the potential of chemical modulator of HH signaling in the treatment of viral diseases. The development of potential new therapies depends on a better understanding of both HH signaling and viral infections to precisely define their interaction along with disease progression.

## Author Contributions

All authors listed have made a substantial, direct, and intellectual contribution to the work, and approved it for publication.

## Conflict of Interest

The authors declare that the research was conducted in the absence of any commercial or financial relationships that could be construed as a potential conflict of interest.

## Publisher’s Note

All claims expressed in this article are solely those of the authors and do not necessarily represent those of their affiliated organizations, or those of the publisher, the editors and the reviewers. Any product that may be evaluated in this article, or claim that may be made by its manufacturer, is not guaranteed or endorsed by the publisher.
